# Association of vitamin D receptor variants with clinical parameters in prostate cancer

**DOI:** 10.1186/s40064-016-2009-8

**Published:** 2016-03-24

**Authors:** Sarah Braga Rodrigues Nunes, Fabrícia de Matos Oliveira, Adriana Freitas Neves, Galber Rodrigues Araujo, Karina Marangoni, Luiz Ricardo Goulart, Thaise Gonçalves Araújo

**Affiliations:** Laboratory of Genetics and Biotechnology, Institute of Genetics and Biochemistry, Federal University of Uberlandia, Campus Patos de Minas, Av. Getúlio Vargas, 230, Sala 206, Patos de Minas, MG 38700-128 Brazil; Faculty of Mathematics, Federal University of Uberlandia, Campus Patos de Minas, Patos de Minas, MG 38700-128 Brazil; Laboratory of Molecular Biology, Federal University of Goias, Goiânia, GO Brazil; Nanobiotechnology Laboratory, Institute of Genetics and Biochemistry, Federal University of Uberlandia, Campus Umuarama, Bloco 2E, Sala 248, Uberlandia, MG 38400-902 Brazil; Department of Medical Microbiology and Immunology, University of California Davis, Davis, CA USA

**Keywords:** Vitamin D receptor, Polymorphisms, Prostate cancer, Prostate-specific antigen

## Abstract

**Purpose:**

Prostate Cancer (PCa) is one of the most common cancers in men and its early detection can provide a high chance of cure. The detection of Vitamin D Receptor (*VDR*) gene polymorphisms may be useful as a molecular indicator of clinical outcome, once VDR is implicated in a wide variety of biological processes including modulation of the immune response and inhibition of cancer cell growth, angiogenesis and metastasis. In this study we explored the Single Nucleotide Polymorphisms (SNPs) *Fok*I, *Bsm*I, *Apa*I and *Taq*I, to evaluate the susceptibility locus for PCa and verify its correlation with clinical parameters.

**Methods:**

*VDR* polymorphisms were detected by PCR followed by Restriction Fragment Length Polymorphism (PCR–RFLP). DNA samples were extracted from peripheral blood of 342 patients: 132 PCa, 41 Benign Prostatic Hyperplasia and 169 young healthy volunteers.

**Results:**

Statistical analysis showed a noteworthy correlation among SNPs and clinical pathological features. CC genotype (*Taq*I) was correlated with the age at diagnosis (>58 years old), and GG (*Bsm*I) was associated to lower Prostate-Specific Antigen (PSA) levels (<10 ng/mL). Moreover, when PCa patients were subgrouped, G allele (*Bsm*I) significantly increased the estimated chance for PSA < 10 ng/mL, and GG/GG genotype (*Bsm*I/*Apa*I) provided a 9.75 fold increased chance of patients with PCa to present lower PSA levels.

**Conclusions:**

The polymorphisms of *VDR* gene showed a genotype-phenotype association and presented new correlations with different parameters as age and PSA levels.

**Electronic supplementary material:**

The online version of this article (doi:10.1186/s40064-016-2009-8) contains supplementary material, which is available to authorized users.

## Background

Prostate cancer (PCa) is the second most common type of cancer in American men. In PCa, structural genomic rearrangements resulting in mutation is associated with the activation of oncogenes leading to tumors development (Prostate Cancer 2015; Wyatt et al. 2013). Early diagnosis and consequently early treatment have resulted in decreased rate of mortality among PCa patients. One of the most common tests used to diagnose PCa was introduced in 1986, when FDA approved the Prostate-Specific Antigen (PSA) for evaluation of the disease progression. In 1994, FDA defined the PSA concentration of 4.0 ng/mlas the upper limit of normal prostate tissue (Barve et al. 2014). However, although it is clinically accepted, the low specificity makes its detection controversial (Cuzick et al. 2014). Therefore, it is highly necessary the development of specific methods that allow early diagnosis of the disease, contributing to the reduction of mortality and providing information on the prognosis before a certain treatment.

Prostate cell division is influenced by two steroid hormones: testosterone and vitamin D. The action of these hormones is mediated by their respective receptors: androgen receptor (AR) and Vitamin D Receptor (VDR) (Jingwi et al. 2015). Prostate epithelial cells express multiple members of nuclear receptor superfamily that regulate proliferation and differentiation of cells in the prostate gland. Their action is disturbed in PCa, presenting molecular alterations and mutations related to the diagnosis of the disease and response to therapy (Gommersall et al. 2004).

VDR is a member of the superfamily of nuclear hormone receptors that regulate gene transcription. The idea that the VDR gene may influence the occurrence of PCa and other diseases is mainly based on the notion that vitamin D is implicated in a wide variety of biological processes including modulation of the immune response and inhibition of cancer cell growth, angiogenesis and metastasis (Li et al. 2015).

The most frequently studied single nucleotide polymorphisms (SNP) in *VDR* are rs1544410, rs731236, rs2228570 and rs7975232, sites for the *Bsm*I, *Taq*I, *Fok*I and *Apa*I restriction enzymes, respectively (Gandini et al. 2014). Such polymorphisms are found in exon 2 (*Fok*I), intron 8 (*Apa*I and *Bsm*I) and exon 9 (*Taq*I). The *Fok*I polymorphism occurs due to a different initiation site associated with a frameshift in the VDR protein (Xu et al. 2014; Mehta et al. 2013). The *Bsm*I and *Apa*I polymorphisms are located into a noncoding region and thus do not affect the quantity, the structure or function of the VDR protein generated. Finally, the *Taq*I is a silent polymorphism caused by the substitution of a cytosine for thymine. As these variants are situated close to the 3ʹ region they can influence the stability of the messenger RNA, altering protein expression (Yang et al. 2014).

Genetic studies have provided excellent opportunities to link molecular insights to epidemiological data. The discovery of genetic variants linked to susceptibility of diseases, mainly the wide variety of tumors, may be the key to improve advances in preventive medicine. Polymorphisms in the VDR gene may be useful to detect individuals with higher risk of disease development, assisting in the early detection and therapy. Furthermore, analysis of haplotypes may be useful to identify groups of linked SNPs, simplifying association analysis and facilitating the understanding of these risk alleles. The aim of this study was to investigate the relationship between the polymorphisms *Fok*I (g.27823C>T), *Bsm*I (g.60890G>A), *Apa*I (g.61888G>T) and *Taq*I (g.61968T>C); alleles namely according to their genomic position (NCBI: #AY342401), and the susceptibility to prostate cancer development as well as their association with clinical parameters.

## Methods

### Study design and sample collection

This work was developed in the Laboratory of Nanobiotechnology of the Federal University of Uberlândia (UFU), approved by the UFU Research Ethics Committee under the approval number 005/2001, together with the Urology Service of the Clinical Hospital of UFU. Peripheral blood samples from Benign Prostatic Hyperplasia (BPH) and PCa were collected before surgery in a vacutainer™ tube containing K_2_ EDTA 7.2 mg, and maintained at 4 °C.

Peripheral blood samples from 342 patients were grouped into three classes: 132 PCa patients, 41 benign prostatic hyperplasia samples and 169 healthy volunteers. Patients were selected by using the following criteria: negative X-rays and bone scan analyses, and rectal examination compatible with organ-confined (i.e. limited to the prostate gland) cancer. Moreover, it was selected BPH patients who were submitted to Transurethral Resection of Prostate (TURP) and PCa patients who were submitted to radical prostatectomy.

PSA levels were obtained through the IMMULITE 1000 System for quantitative detection (Siemens Healthcare Diagnostics Inc.), considering normal values between 0 and 4.0 ng/mL. DNA was extracted from leukocytes according to protocol previously published elsewhere (Sambrook et al. 1989) and the concentration and quality were obtained spectrophotometrically by the absorbance readings at 260 and 280 nm.

### Single nucleotide polymorphisms screening

**Fig. 1:**
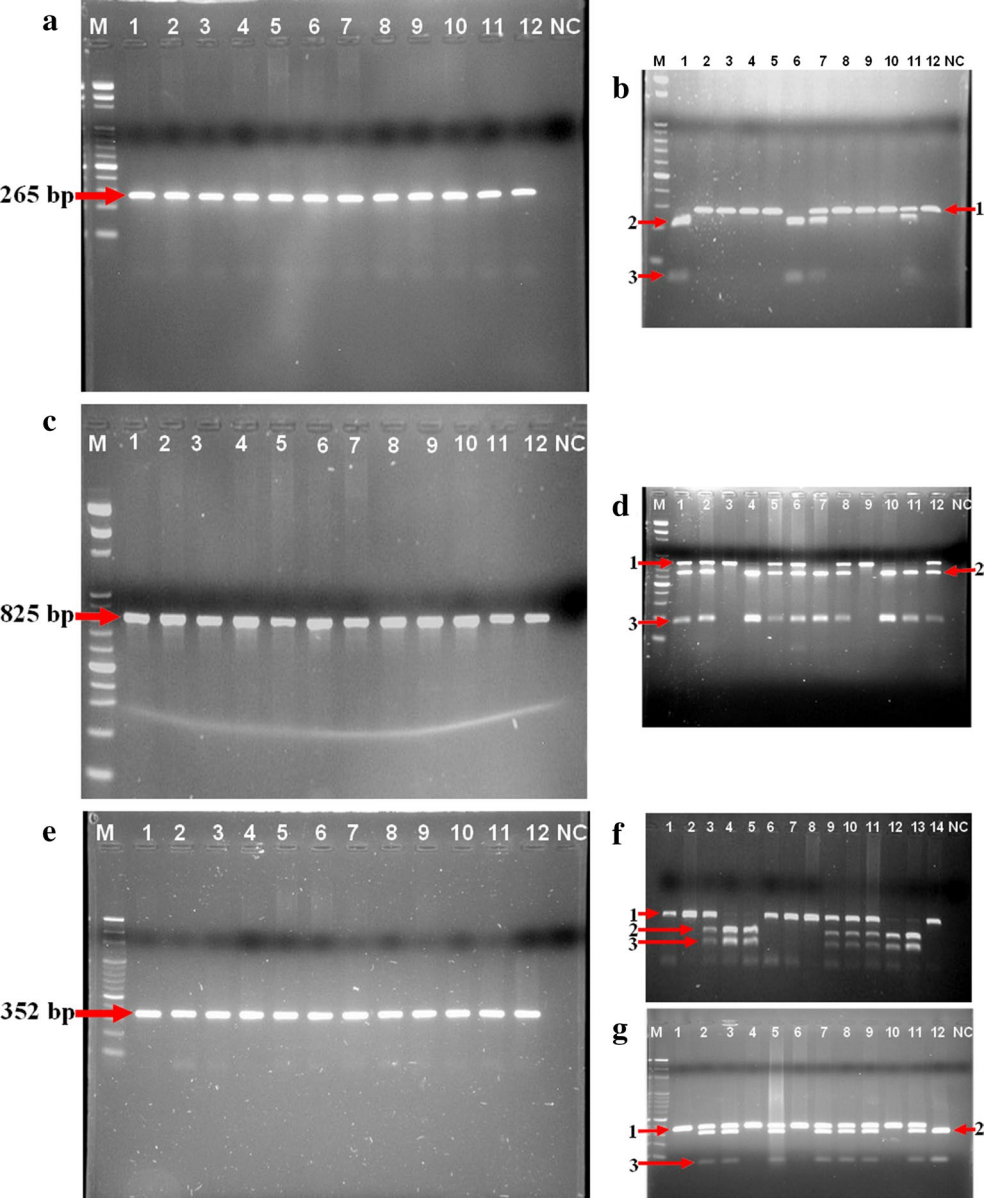
Amplification of *VDR* gene and restriction endonuclease digestion pattern for *FokI*, *BsmI*, *ApaI* and *TaqI* polymorphims. The 265 bp fragment (**a**) was digested by *Fok*I enzyme (**b**) generating two fragments, 196 (2) and 69 bp (3) for T allele. C allele, containing 265 bp (1), represents the absence of restriction site. The 825 bp fragment (**c**) was digested by *Bsm*I enzyme (**d**) presenting two fragments, 650 (2) and 175 bp (3) for G allele, and 825 bp (1) for A allele (no restriction site). The amplicon of 325 bp (**e**) was used for detection of *ApaI* and *TaqI* polymorphims. For *ApaI*, the 352 bp fragment (1) represents T allele, and the 214 bp (2) and 18 bp (3) fragments indicate the presence of G allele (**f**). Finally, after digestion with *TaqI* enzyme (**g**) it was verified two fragments, 293 (2) and 59 bp (3) for C allele and a unique fragment of 352 bp (1) for T allele. M: 100 bp DNA ladder. *Lanes* 1–4: represent PCa patients; *Lanes* 5–8: represent BPH patients; *Lanes* 9–12: represent healthy volunteers. *NC* negative control

The SNPs with restriction site for *FokI*, *BsmI*, *ApaI* e *TaqI* enzymes presented in *VDR* gene were detected by PCR followed by restriction fragment length polymorphism (PCR–RFLP) method (Fig. 1). To identify the *FokI* mutation, we amplified the 265-bp PCR fragment (Fig. 1a) and performed an endonuclease *FokI* digestion. In the absence of the mutation, cleaved fragments of 196 and 69-bp were detected (Fig. 1b). For the *BsmI* mutation, the 825-bp amplified fragment (Fig. 1c) was digested by *BsmI*, and in the absence of the mutation, cleaved fragments of 650 and 175-bp were detected (Fig. 1d). To detect the *ApaI* mutation, the 352-bp (Fig. 1e) amplified fragment was digested by *ApaI*. If the fragment had this mutation, cleaved fragments of 214 and 138-bp could be obtained (Fig. 1f). For the *TaqI* mutation, the 352-bp amplified fragment was digested by *TaqI*, and cleaved fragments of 293 and 59-bp could be obtained if the fragment had this mutation (Fig. 1g). Each fragment was detected in a 2.5 % agarose gel stained with ethidium bromide. Primers designed for fragments amplification are described in Table 1.

**Table 1:** Oligonucleotide sequences for DNA amplification

SNPs	Primer sequence 5′–3′ (forward/reverse)	Position in database sequence	Amplicon (pb)
*rs731236 (Taq*I)	CAGAGCATGGACAGGGAGCAAG	exon 9	352
	GGTGGCGGCAGCGGATGTACGT		
*rs7975232 (Apa*I)	CAGAGCATGGACAGGGAGCAAG	intron 8	352
	GGTGGCGGCAGCGGATGTACGT		
*rs15444410 (Bsm*I)	CAACCAAGACTCAAGTACCGCGTCAGTGA	intron 8	823
	AACCAGCGGAAGAGGTCAAGGG		
*rs2228570 (Fok*I)	AGCTGGCCCTGGCACTGACTCTGCTCT	exon 2	265
	ATGGAAACACCTTGCTTCTTCTCCCTC		

*SNP* single nucleotide polymorphism

### Statistical analyses

Allele frequency analysis and tests of deviation from Hardy–Weinberg equilibrium were carried out using the GraphPad Prism 5 (GraphPad Software, La Jolla, CA, USA). Chi square analyses were performed to compare genotypic and allelic frequencies for the average of clinical parameters, such as: age, PSA serum levels, TNM (Tumor-Node-Metastasis), adenocarcinoma histopathological staging and Gleason score. The *odds ratio* (OR) was determined to verify the risk of prostate cancer development. P values <0.05 were considered statistically significant.

Associations between SNPs and clinical data were performed with the contingency coefficient C by using BioEstat 5.3 software.

## Results

Table 2 shows *VDR* polymorphisms with their respective genotypes, and allelic frequencies in PCa group (n = 132), BPH (n = 41) and population control (n = 169). All genotypic frequencies were in Hardy–Weinberg equilibrium, but no differences in genotypes and alleles were observed between the three groups. Parameters as age and PSA levels were not different comparing PCa and BPH groups. We observed strong linkage disequilibrium between *Taq*I, *Bsm*I and *Apa*I polymorphisms located in exon 8 and exon 9 of *VDR* gene (Table 3).

**Table 2:** Genotypic and allelic frequencies for VDR gene polymorphisms and clinical parameters of patients with prostate cancer, benign prostatic hyperplasia and healthy volunteers

SNP	PCa (N = 132)			BPH (N = 41)			Healthy volunteers (N = 169)	
	N (%)	Age*	PSA*	N (%)	Age*	PSA*	N (%)	Age*
*Fok*I								
CC	54.00 (40.91)	65.08 (8.79)	14.08 (18.35)	18.00 (43.90)	66.25 (10.67)	18.30 (46.56)	79.00 (46.75)	23.00 (4.57)
CT	62.00 (46.97)	67.51 (8.41)	19.21 (32.75)	19.00 (46.34)	70.00 (10.59)	12.75 (16.73)	76.00 (44.97)	22.98 (4.31)
TT	16.00 (12.12)	65.53 (11.29)	50.68 (123.35)	4.00 (9.76)	72.33 (5.77)	8.27 (2.22)	14.00 (8.38)	22.86 (6.18)
Alleles C/T	170/94 (64.39/35.61)			55/27 (67.07/32.93)			234/104 (69.23/30.77)	
^§^P_HWE_	0.78			0.75			0.47	
Pχ^2^	PCa x BPH = 0.90			PCa x Healthy = 0.42			BPH x Healthy = 0.93	
*Bsm*I								
AA	14.00 (10.61)	64.31 (9.66)	30.11 (57.48)	5.00 (12.20)	71.00 (8.21)	3.53 (2.40)	28.00 (16.57)	23.37 (4.50)
AG	63.00 (47.73)	67.69 (8.24)	17.09 (22.57)	25.00 (60.98)	66.80 (11.25)	10.41 (14.68)	70.00 (41.42)	22.93 (4.78)
GG	55.00 (41.67)	65.31 (9.44)	21.49 (61.54)	11.00 (26.86)	71.45 (8.71)	26.14 (55.23)	71.00 (42.01)	22.91 (4.15)
Alleles A/G	91/173 (34.47/65.53)			35/47 (42.68/57.32)			126/212 (37.28/62.72)	
^§^P_HWE_	0.52			0.11			0.14	
Pχ^2^	PCa x BPH = 0.23			PCa x Healthy = 0.28			BPH x Healthy = 0.08	
*Apa*I								
TT	49.00 (37.12)	65.74 (9.22)	19.98 (36.07)	18.00 (43.90)	66.56 (10.78)	9.72 (17.38)	51.00 (30.18)	22.36 (4.94)
TG	59.00 (44.70)	65.44 (9.29)	25.48 (63.84)	16.00 (39.02)	69.57 (10.50)	8.45 (6.10)	89.00 (52.66)	22.91 (4.33)
GG	24.00 (18.18)	69.30 (7.18)	10.66 (12.26)	7.00 (17.07)	71.14 (9.23)	37.11 (68.48)	29.00 (17.16)	24.77 (3.94)
Alleles T/G	157/107 (59.47/40.53)			52/30 (63.41/36.59)			191/147 (56.51/43,49)	
^§^P_HWE_	0.40			0.31			0.35	
Pχ^2^	PCa x BPH = 0.73			PCa x Healthy = 0.35			BPH x Healthy = 0.21	
*Taq*I								
TT	60.00 (45.45)	66.09 (8.94)	20.77 (60.47)	13.00 (31.71)	70.75 (8.66)	23.79 (53.24)	71.00 (42.01)	23.33 (4.01)
TC	62.00 (46.97)	66.93 (8.92)	17.59 (21.26)	23.00 (56.10)	66.38 (11.44)	10.69 (15.05)	75.00 (44.38)	22.90 (5.00)
CC	10.00 (7.58)	63.25 (9.78)	37.62 (72.71)	5.00 (12.20)	73.50 (6.24)	6.03 (2.65)	23.00 (13.61)	22.40 (4.47)
Alleles T/C	182/82 (68.94/31.06)			49/33 (59.76/40.24)			217/121 (64.20/35.80)	
^§^P_HWE_	0.27			0.29			0.65	
Pχ^2^	PCa x BPH = 0.26			PCa x Healthy = 0.25			BPH x Healthy = 0.39	

Pχ^2^: P value of Chi square test

*SNP* single nucleotide polymorphism

* Mean (±SD)

^§^P_HWE_: Hardy–Weinberg equilibrium

**Table 3:** Contingency table for analysis of linkage disequilibrium between *Apa*I*, Bsm*I and *Taq*I polymorphisms

*Taq*I versus *Bsm*I^a^	AA	AG	GG
TT	1 (0.29)	19 (5.56)	124 (36.26)
TC	11 (3.22)	137 (40.06)	12 (3.51)
CC	35 (10.23)	2 (0.58)	1 (0.29)

*Apa*I versus *Taq*I^b^	TT	TC	CC
TT	21 (6.14)	60 (17.54)	37 (10.82)
GT	65 (19.01)	99 (28.95)	0
GG	58 (16.96)	1 (0.29)	1 (0.29)

*Bsm*I versus *Apa*I^c^	TT	GT	GG
AA	46 (13.45)	1 (0.29)	0
AG	55 (16.08)	102 (29.82)	1 (0.29)
GG	17 (4.97)	61 (17.84)	59 (17.25)

^a^ *Taq*I versus *Bsm*I: χ^2^ = 426.57, P < 0.0001*

^b^ *Apa*I versus *Taq*I: χ^2^ = 158.77, P < 0.0001*

^c^ *Bsm*I versus *Apa*I: χ^2^ = 188.96, P < 0.0001*

* Significant data

Combined genotypes analysis demonstrated a statistically significant correlation between genotypes and the parameters of diagnosis. The estimated OR for BPH occurrence compared to healthy volunteers for AG/TT genotype (*Bsm*I/*Apa*I) was 3.90 (95 % CI 1.19–12.84, P = 0.04). The OR for AG/TT (*Bsm*I/*Taq*I) comparing PCa group and healthy volunteers was 3.30 (95 % CI 1.09–10.00, P = 0.05). GG/TT (*Bsm*I*/Apa*I) genotypes were responsible for providing higher chances to healthy volunteers to develop PCa (OR 3.54, 95 % CI 1.08–11.57, P = 0.007).

Although not significant, a 2.37-fold chance for the occurrence of hyperplasia was observed in heterozygous individuals AG/TC (*Bsm*I*/Taq*I) compared to healthy volunteers (95 % CI 1.05–5.38, P = 0.06) (Table 4). Genotype-level associations between diagnosis, clinical parameters and the four vitamin D variants considering Coefficient C are displayed in Table 5. TT genotype (*Taq*I) was more frequent in patients with lower PSA levels (30.21 %; P = 0.09), and GG genotypes (*Apa*I) seems to be associated to older men in the BPH group (P = 0.07).

**Table 4:** Combined genotypic frequency for *Apa*I*, Taq*I and *Bsm*I polymorphisms in *VDR* gene

Genotypes	PCa X BPH		PCa X healthy volunteers		BPH X healthy volunteers	
	OR (95 % CI)	P	OR (95 % CI)	P	OR (95 % CI)	P
*Bsm I* - *Apa I*						
AATT	1.00	Reference	1.00	Reference	1.00	Reference
AGTT	0.66 (0.19–2.24)	0.71	2.57 (1.06–6.26)	0.06	3.90 (1.19–12.84)	0.04*
GGGT	1.88 (0.43–8.22)	0.64	1.13 (0.49–2.61)	0.95	0.60 (0.15–2.45)	0.72
AGGT	1.13 (0.34–3.79)	0.91	1.41 (0.65–3.04)	0.49	1.25 (0.40–3.91)	0.93
GGGG	1.17 (0.31–4.42)	0.92	1.53 (0.66–3.57)	0.44	1.30 (0.37–4.60)	0.93
GGTT	ND	ND	3.54 (1.08–11.57)	0.07	ND	ND
*Apa I* - *Taq I*						
TTTT	1.00	Reference	1.00	Reference	1.00	Reference
TTTC	0.69 (0.16–2.97)	0.89	0.85 (0.28–2.56)	0.99	1.22 (0.26–5.68)	0.90
GTTT	1.71 (0.33–8.93)	0.85	0.44 (0.15–1.30)	0.22	0.26 (0.05–1.42)	0.26
GTTC	0.77 (0.18–3.25)	1.00	0.41 (0.14–1.16)	0.15	0.53 (0.12–2.35)	0.67
TTCC	0.68 (0.12–3.83)	1.00	0.28 (0.08–0.92)	0.07	0.41 (0.07–2.27)	0.57
GGTT	1.09 (0.23–5.19)	0.77	0.55 (0.18–1.63)	0.41	0.50 (0.10–2.51)	0.69
*Bsm I* - *Taq I*						
GGTT	1.00	Reference	1.00	Reference	1.00	Reference
AGTC	0.45 (0.20–1.06)	0.10	1.07 (0.64–1.82)	0.89	2.37 (1.05–5.38)	0.06
AATC	ND	ND	1.15 (0.33–3.97)	0.92	ND	ND
AACC	0.47 (0.12–1.83)	0.47	0.56 (0.24–1.33)	0.27	1.20 (0.34–4.21)	0.96
AGTT	1.25 (0.24–6.48)	0.89	3.30 (1.09–10.00)	0.05*	2.64 (0.45–15.49)	0.58
GGTC	ND	ND	1.93 (0.58–6.53)	0.44	ND	ND
AGCC	ND	ND	1.38 (0.08–22.54)	0.62	ND	ND
HAPLOTYPES						
*Bsm I* - *Apa I*						
AT	1.00	Reference	1.00	Reference	1.00	Reference
GT	1.28 (0.71–2.32)	0.51	1.08 (0.72–1.62)	0.78	0.85 (0.48–1.51)	0.67
GG	1.43 (0.76–2.66)	0.34	0.92 (0.61–1.38)	0.75	0.64 (0.35–1.18)	0.20
AG	1.29 (0.59–2.78)	0.66	0.95 (0.57–1.58)	0.94	0.74 (0.35–1.56)	0.54
*Apa I* - *Taq I*						
TT	1.00	Reference	1.00	Reference	1.00	Reference
TC	0.82 (0.45–1.49)	0.61	0.89 (0.59–1.33)	0.63	1.09 (0.61–1.95)	0.90
GT	1.16 (0.62–2.15)	0.77	0.84 (0.57–1.24)	0.43	0.73 (0.40–1.33)	0.38
GC	0.80 (0.38–1.71)	0.71	0.75 (0.45 to1.25)	0.33	0.94 (0.45–1.94)	0.99
*Bsm I* - *Taq I*						
AT	1.00	Reference	1.00	Reference	1.00	Reference
GC	0.94 (0.49–1.81)	0.98	0.93 (0.57–1.50)	0.85	1.0 (0.52–1.88)	0.90
GT	1.30 (0.72–2.34)	0.48	0.94 (0.62–1.41)	0.83	0.72 (0.40–1.29)	0.34
AC	0.93 (0.49–1.77)	0.96	0.78 (0.50–1.24)	0.35	0.84 (0.45–1.56)	0.69

*ND* no data, *CI* confidence interval

* Significant data

**Table 5:** Coefficient C for *VDR* polymorphisms and clinical data

SNPs	PCa						BPH		
	Age < 58 years old	Age ≥ 58 years old	P	PSA < 10 ng/mL	PSA ≥ 10 ng/mL	P	Age < 58 years old	Age ≥ 58 years old	P
*Taq*I									
TT	2 (1.82)	6 (5.45)	<0.0001*	29 (30.21)	15 (15.63)	0.09	0	4 (10.81)	0.0004*
TC	38 (34.55)	8 (7.27)		22 (22.92)	23 (23.96)		15 (40.54)	6 (16.22)	
CC	12 (10.91)	44 (40.00)		2 (2.08)	5 (5.21)		1 (2.70)	11 (29.73)	
*Bsm*I									
AA	3 (2.50)	10 (8.33)	0.35	2 (2.08)	9 (9.38)	0.02*	0	4 (10.81)	0.27
AG	7 (5.83)	48 (40.00)		23 (23.96)	20 (20.83)		6 (16.22)	16 (43.24)	
GG	12 (10.00)	40 (33.33)		28 (29.17)	14 (14.58)		1 (2.70)	10 (27.03)	
*Apa*I									
GG	1 (0.83)	22 (18.33)	0.71	18 (18.75)	19 (19.79)	0.30	1 (2.70)	6 (16.22)	0.07
GT	14 (11.67)	40 (33.33)		21 (21.88)	18 (18.75)		2 (5.41)	12 (32.43)	
TT	7 (5.83)	36 (30.00)		14 (14.58)	6 (6.25)		4 (10.81)	12 (32.43)	

*SNP* single nucleotide polymorphism

* Significant data

In a significant manner, CC polymorphism (*Taq*I) was associated to men over 58 years of age in PCa (P < 0.0001) and BPH (P = 0.0004) groups. In addition, GG genotype (*Bsm*I) was associated to lower PSA levels (PSA < 10 ng/mL) in PCa group (P = 0.02), which is considered a prognostic factor.

Additional analyses were performed to clarify the effect of genotypes and haplotypes in PCa group and their correlation with clinical pathological features (Table 6 and Additional file 1 for complete data). All polymorphism were analyzed and those which were not significant were omitted. The data indicated that patients containing the GG genotype (*Bsm*I) have a prevalence 9.00 times higher to present PSA levels <10 ng/mL (95 % CI 1.71–47.39, P = 0.01). Moreover, G allele (*Bsm*I) significantly increased the chances of lower PSA levels in the recessive (OR 6.75, 95 % CI 1.37–33.18, P = 0.02), and dominant models (OR 2.32, 95 % CI 1.01–5.35, P = 0.07). It is also observed that the T allele of the polymorphism *Taq*I in recessive model was associated to PSA < 10 ng/mL in PCa group (P = 0.08), but it was not significant.

**Table 6:** *VDR* polymorphims sub grouping in PCa cases according to clinical parameters

Polymorphisms	PSA N (%)				Gleason N (%)				TNM N (%)			
Genotypes	<10 ng/mL	≥10 ng/mL	*Odds* (95 % CI)	P	<7	≥7	*Odds* (95 % CI)	P	T1-T2	T3	*Odds* (95 % CI)	P
*Fok*I												
CC	21 (21.88)	16 (16.67)	1.00		26 (24.53)	18 (16.98)	1.00		16 (22.22)	16 (22.22)	1.00	
CT	25 (26.04)	24 (25.00)	0.79 (0.34–1.87)	0.76	27 (25.47)	21 (19.81)	0.89 (0.39–2.04)	0.95	24 (33.33)	8 (11.11)	3.00 (1.04–8.65)	0.07
TT	7 (7.29)	3 (3.13)	1.78 (0.40–7.97)	0.69	7 (6.60)	7 (6.60)	0.69 (0.21–2.32)	0.77	5 (6.94)	3 (4.17)	1.67 (0.34–8.18)	0.81
*Bsm*I												
AA	2 (2.08)	9 (9.38)	1.00		8 (7.55)	4 (3.77)	1.00		8 (11.11)	2 (2.78)	1.00	
AG	23 (23.96)	20 (20.83)	5.18 (1.00–26.82)	0.08	25 (23.58)	21 (19.81)	0.60 (0.16–2.26)	0.66	16 (22.22)	11 (15.28)	0.36 (0.07–2.05)	0.43
GG	28 (29.17)	14 (14.58)	9.00 (1.71–47.39)	0.01*	27 (25.47)	21 (19.81)	0.64 (0.17–2.43)	0.74	21 (29.17)	14 (19.44)	0.38 (0.07–2.03)	0.43
Genotypes in pairs												
*Bsm*I - *Apa*I												
AATT	2 (2.11)	9 (9.47)	1.00		8 (8.25)	4 (4.12)	1.00		8 (12.70)	2 (3.17)	1.00	
AGTT	10 (10.53)	7 (7.37)	6.43 (1.05–39.33)	0.08	9 (9.28)	6 (6.19)	0.75 (0.15–3.65)	0.97	7 (11.11)	4 (6.35)	0.44 (ND)	0.73
GGGT	9 (9.47)	5 (5.26)	8.10 (1.23–53.20)	0.06	4 (4.12)	6 (6.19)	0.33 (0.06–1.91)	0.41	4 (6.35)	4 (6.35)	0.25 (ND)	0.40
AGGT	12 (12.63)	13 (13.68)	4.15 (0.74–23.23)	0.19	15 (15.46)	15 (15.46)	0.50 (0.12–2.02)	0.52	9 (14.29)	6 (9.52)	0.38 (0.06–2.41)	0.54
GGGG	13 (13.68)	6 (6.32)	9.75 (1.59–59.70)	0.02*	12 (12.37)	7 (7.22)	0.86 (0.19–3.92)	0.85	7 (11.11)	4 (6.35)	0.44 (ND)	0.73
GGTT	6 (6.32)	3 (3.16)	9.00 (ND)	0.08	4 (4.12)	6 (6.19)	0.33 (0.06–1.91)	0.41	4 (6.35)	4 (6.35)	0.25 (ND)	0.40
Dominant model												
*Bsm*I												
AA + AG	25 (26.04)	29 (30.21)	1.00		33 (31.43)	25 (23.81)	1.00		24 (33.33)	13 (18.06)	1.00	
GG	28 (29.17)	14 (14.58)	2.32 (1.01–5.35)	0.07	26 (24.76)	21 (20.00)	0.94 (0.43–2.04)	0.97	21 (29.17)	14 (19.44)	0.81 (0.31–2.11)	0.86
Recessive model												
*Fok*I												
CC	21 (21.88)	16 (16.67)	1.00		25 (23.81)	18 (17.14)	1.00		16 (22.22)	16 (22.22)	1.00	
CT + TT	32 (33.33)	27 (28.13)	0.90 (0.40–2.07)	0.98	34 (32.38)	28 (26.67)	0.87 (0.40–1.92)	0.89	29 (40.28)	11 (15.28)	2.64 (0.99–7.03)	0.09
*Taq*I												
TT	29 (30.21)	15 (15.63)	1.00		27 (25.71)	22 (20.95)	1.00		21 (29.17)	17 (23.61)	1.00	
TC + CC	24 (25.00)	28 (29.17)	0.44 (0.19–1.02)	0.08	32 (30.48)	24 (22.86)	1.09 (0.50–2.35)	0.99	24 (33.33)	10 (13.89)	1.94 (0.73–5.16)	0.27
*Bsm*I												
AA	2 (2.08)	9 (9.38)	1.00		8 (7.62)	4 (3.81)	1.00		8 (11.11)	2 (2.78)	1.00	
AG + GG	51 (53.13)	34 (35.42)	6.75 (1.37–33.18)	0.02*	51 (48.57)	42 (40.00)	0.61 (0.17–2.16)	0.64	37 (51.39)	25 (34.72)	0.37 (0.07–1.89)	0.38

*ND* no data, *CI* confidence interval

* Significant data

Individuals displaying GG/GG (*Bsm*I/*Apa*I) are 9.75 times more likely to present low levels of PSA (95 % CI 1.59–59.70, P = 0.02). The combination of *Bsm*I and *Apa*I variants showed that AG/TT (OR 6.43, 95 % CI 1.05–39.33, P = 0.08), and GG/GT (OR 8.10, 95 % CI 1.23–53.20, P = 0.06) genotypes provide an increased chance of presenting PSA < 10 ng/mL in PCa patients (Table 6).

Evaluating pathological data, CT (*Fok*I) individuals showed a 3.00-fold increased risk for confined PCa tumors (T1–T2) compared to disease progression (T3) (95 % CI 1.04–8.65, P = 0.07). Moreover, evaluating the recessive model, the T allele from the same SNP presented a 2.64-fold decreased risk to T3 tumors development (95 % CI 0.99–7.03 P = 0.09).

## Discussion

While VDR regulates numerous genes across the genome, much remains to be learned about pathways and cellular interactions. VDR functions have a broad impact, contributing to cancer development once it regulates cell proliferation and cell cycle control. A better understanding of the genetic factors related to this receptor may facilitate the development of improved strategies for the diagnostic and prognostic of prostate cancer (Saccone et al. 2015). In the analyses presented herein, we did not find associations between prostate cancer risk and *Fok*I, *Bsm*I, *Apa*I and *Taq*I polymorphisms, when considered individually.

A functional activity of the *Fok*I polymorphism and its involvement with the incidence of prostate cancer has already been reported (John et al. 2005). Although the SNP *Fok*I seems to be functional and the 424 amino acids (aa) *VDR* variant is somewhat more active than the 427 aa in terms of its transactivation capacity as a transcription factor, no association between this mutation, prostate cancer and BPH has been reported to date (Hayes et al. 2005; Zeng et al. 2014).

*VDR* gene polymorphisms may affect the binding of 1,25-(OH)_2_D_3_ to its receptor and thereby compromise the anti-proliferative effects of vitamin D. Various polymorphisms in 3′ *cluster* of the gene have been identified and it is not known whether these represent functional genetic differences or just mark the disease risk alleles (Whitfield et al. 2001). Although genetic variants in the VDR itself do not appear to be linked to 25(OH)D levels, they exert an influence in VDR expression and function, especially considering mRNA stability. Through the 3′ untranslated region, the SNPs *Bsm*I, *Apa*I and *Taq*I are silent polymorphisms.

Age is a well-established risk factor that contributes to the etiology of PCa. The chance of PCa occurrence rises after 50-years of age, and about 6 cases in 10 are diagnosed in men aged 65 or older (Prostate Cancer 2015). It has been reported that the onset of disease at a young age has correlated with more aggressive tumor types and subsequent mortality, contributing to poorer prognosis (Bratt et al. 1998). In patients with PCa and BPH, we found an association between *Taq*I polymorphism (CC genotype) and increased age (>58-year-old) suggesting that the detection of this polymorphism could help to determine prognosis.

The *Bsm*I polymorphism in the *VDR* gene has been described as a possible genetic marker for different clinical conditions such as type 1 diabetes, obesity and some types of cancers (Cavalcante et al. 2015). A previous study investigating the *Bsm*I polymorphism in multiple sclerosis showed that the G allele contributed to providing a protective effect, while the A allele showed a positive association with the pathology (Narooie-Nejad et al. 2015). Moreover, functional data on VDR level in peripheral blood mononuclear cells of healthy subjects demonstrated that homozygosity for *Bsm*I ‘A’ allele and *Taq*I ‘C’ allele is associated with lower levels of VDR protein (Saccone et al. 2015; Selvaraj 2009). In our work, G allele was correlated to lower levels of PSA in PCa patients, which also represents a protective effect.

Vitamin D also impacts prostate cancer, regulating androgen-responsive and androgen-metabolizing genes. Androgens act through their receptor to regulate prostate growth and play an important role in the development and progression of PCa (Krishnan et al. 2003). A crosstalk between VDR and the Androgen Receptor (AR) has already been suggested, since LNCaP cells have intensively responded to dihydrotestosterone in the presence of 1,25-(OH)_2_D_3_. Furthermore, 1,25-(OH)_2_D_3_ and dihydrotestosterone exhibit synergistic interaction to regulate the LNCaP cell proliferation and PSA secretion in a heterologous up-regulation of AR by 1,25-(OH)_2_D_3_ (Zhao et al. 1997). Considering *Bsm*I polymorphisms, our results demonstrating the association between G allele and lower PSA levels highly support the idea that VDR and AR share the same coregulators in a crosstalk between both receptors (Williams et al. 2004). The overexpression of AR in PC-3 cell line and the activation of AR in LNCaP cells can suppress VDR transactivation corroborating with these two-way molecular interaction, which may be coordinated by ARA70 coregulator (Ting et al. 2005).

The PSA expression is rigidly controlled by androgens via the AR (Young et al. 1991). Partin et al. (1993) demonstrated that patients with pre-operative serum PSA concentrations higher than 10.0 ng/mL are at a statistically increased risk of PCa recurrence. Additionally, PSA levels higher than 2.0 ng/mL during the year before the diagnosis increased the risk of mortality, despite undergoing radical prostatectomy (D’Amico et al. 2004).

It has been shown that the A allele is protective for men with locally advanced disease, and is correlated with a poorer prognosis among men with organ-confined disease (Williams et al. 2004). In our study the G allele may be associated with higher levels of VDR protein, lower levels of PSA and contributes to organ-confined disease, decreasing the possibility of PCa recurrence. Based on these findings, we hypothesize that *VDR* polymorphisms associated to PSA levels may be useful as a prognostic factor.

Strong linkage disequilibrium between the *Bsm*I*, Apa*I*, and Taq*I polymorphisms have been reported and are linked to the risk of prostate cancer (Mikhak et al. 2007). In fact, polymorphisms tend to be inherited, and their various possible combinations may have a significant association with the disease phenotype (Jingwi et al. 2015). In our study, this linkage disequilibrium was confirmed (P < 0.0001). Haplotypes G/T (SNPs *Apa*I/*Taq*I), A/T and G/G (SNPs *Bsm*I/*Apa*I), T/G and C/A (SNPs *Taq*I/*Bsm*I) were the most frequent in our population, and the four polymorphisms were found in the Hardy–Weinberg equilibrium.

Our data demonstrated that AG/TT genotype (*Bsm*I/*Apa*I) conferred higher risk of hyperplasic disease development. This data is supported by a previous study in which *Taq*I and *Bsm*I variants were associated with the susceptibility to the development of benign prostatic hyperplasia within an Indian population (Manchanda et al. 2010). Furthermore, studies have shown that the *Bsm*I and *Taq*I variants can play a significant role in the development of prostate cancer (Taylor et al. 1996; Huang et al. 2004). In our study, the AG/TT genotype (*Bsm*I*/Taq*I) was responsible for an increased chance for PCa occurrence compared to controls.

These genetic polymorphisms associated with PCa have been extensively studied generating contradictory results (Jingwi et al. 2015; Xu et al. 2014). Here we demonstrated the association between VDR polymorphism and critical clinical parameters such as PSA levels and age.

## Conclusion

This is the first study that describes an association between VDR polymorphisms and clinical data. We found an association between *Taq*I polymorphism (CC genotype) and increased age (>58-year-old) in patients with PCa and BPH. Besides, *Bsm*I G allele was correlated to lower levels of PSA in PCa patients.

Future studies with a large population cohort focused on genotyping additional polymorphisms to capture more of the variations in the *VDR* gene, and haplotype analysis to elucidate the role of the *VDR* gene as a prostate cancer risk factor may benefit the expansion of significant data. Our work demonstrated a correlation between age and PSA in PCa, opening new perspectives on using polymorphic markers clinically.

## Additional file

10.1186/s40064-016-2009-8 *ApaI*, *FokI*, *TaqI* and *BsmI* polymorphims sub grouping in PCa cases according toclinical parameters
