# Supplementary figures and images for: Association of vitamin D receptor variants with clinical parameters in prostate cancer
Source: Springerplus. 2016 Mar 24;5:364. doi: 10.1186/s40064-016-2009-8 (PMC4805678; doi:10.1186/s40064-016-2009-8)

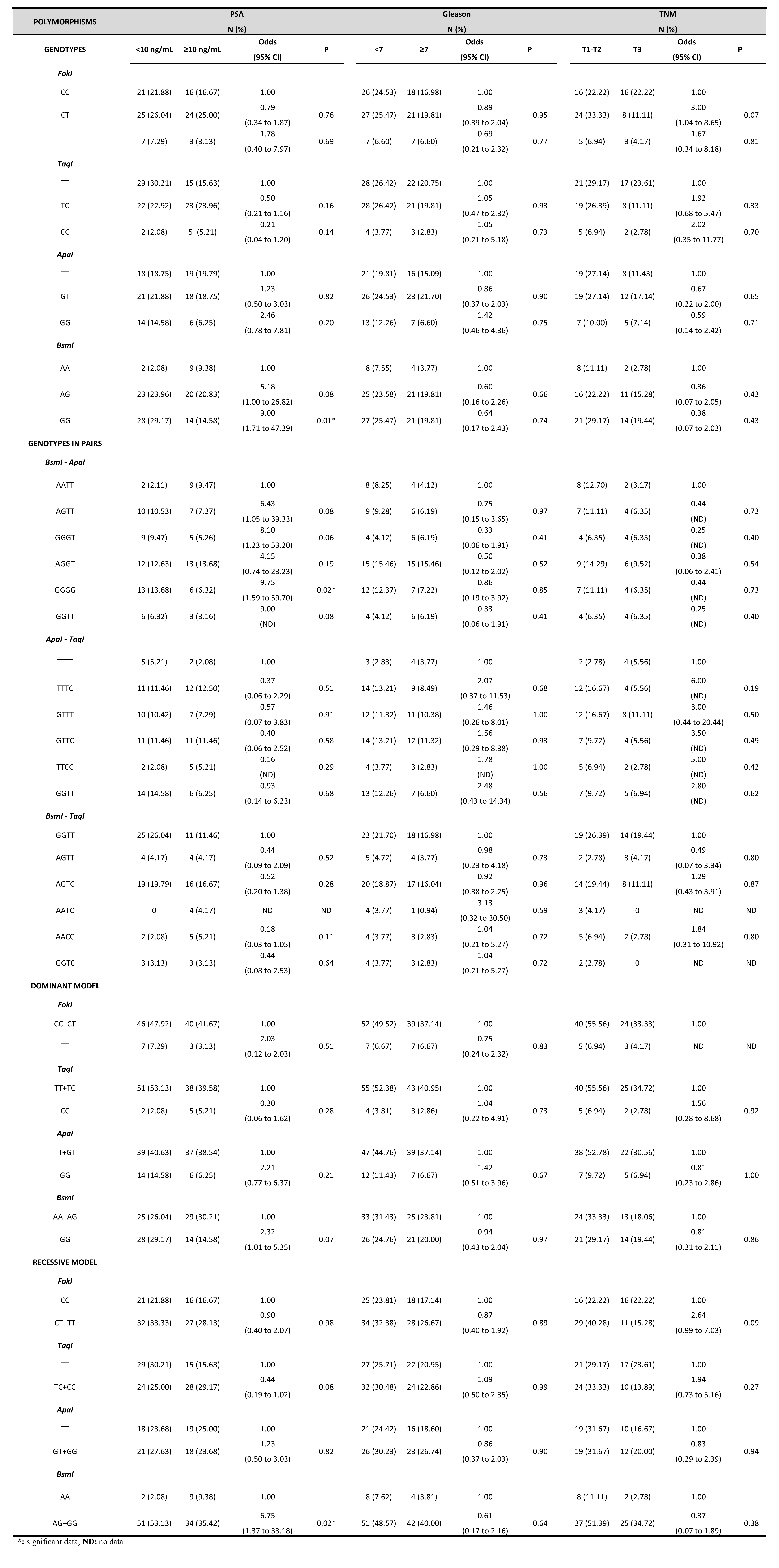

Supplement: Supplementary file 1 — 10.1186/s40064-016-2009-8 ApaI, FokI, TaqI and BsmI polymorphims sub grouping in PCa cases according toclinical parameters [file 40064_2016_2009_MOESM1_ESM.tif]
